# Machine learning approaches for forecasting compressive strength of high-strength concrete

**DOI:** 10.1038/s41598-025-10342-1

**Published:** 2025-07-15

**Authors:** Mohammed Shaaban, Mohamed Amin, S. Selim, Islam M. Riad

**Affiliations:** 1 Civil Engineering Department, Faculty of Engineering, Delta University for Science and Technology, International Coastal Road, Gamasa, Egypt; 2 Civil and Architectural Constructions Department, Faculty of Technology and Education, Suez University, Suez, Egypt; 3 Civil Engineering Department, Mansoura Higher Institute of Engineering and Technology, Mansoura, Egypt; 4 Structural Engineering Department, Faculty of Engineering, Mansoura University, Mansoura, Egypt

**Keywords:** Python, Prediction models, Machine learning, High strength concrete, Civil engineering, Structural materials

## Abstract

Identifying the mechanical properties of High Strength Concrete (HSC), particularly compressive strength, is critical for safety purposes. Concrete compressive strength is determined by using laboratory experiments, which are costly and time-consuming. Artificial intelligence (AI) methods reduce time and money. This research proposes a machine learning (ML) model using the Python programming language to predict the compressive strength of HSC. The dataset used for the models was obtained from original experimental tests. Important parameters, namely cement content, silica fume, water, superplasticizer, sand, gravel, and curing age, were taken as input to predict the output, which was the compressive strength. Various regression models were investigated for the prediction of outcome compressive strength. To optimize the models, hyperparameters were tuned, and measures such as Mean Absolute Error (MAE), Mean Squared Error (MSE), and R-squared were used for evaluation. XGBoost (*R*^2^ ≈ 0.94) outperformed other models, demonstrating ML’s potential for HSC strength prediction and demonstrated that Python can be successfully applied to establish accurate and reliable prediction models.

**Supplementary Information:**

The online version contains supplementary material available at 10.1038/s41598-025-10342-1.

## Introduction

Concrete is the most frequently utilized building material in the world due to its several advantages over other materials, including integrity, durability, modularity, and cost. Concrete is composed of a variety of elements such as coarse aggregate, fine aggregate, water, and binder, among others. These components are randomly distributed over the entire concrete matrix. For the effective evaluation of the performance of concrete according to the advanced design technologies, its mechanical properties must be examined. One of its outstanding mechanical features is compressive strength. Concrete compressive strength is a critical parameter in the design and study of concrete structures because it is directly related to structural safety and is required in the performance determination of structures throughout their entire life cycle, from new structural design to old structural assessment. This unique characteristic of concrete can be altered by several elements, such as particle size, water-to-cement ratio, waste makeup, and chemical usage^1–4^.

In general, physical experiments are the most straightforward technique to determine concrete’s compressive strength. Typically, cubic or cylindrical specimens were made according to a specific planned mixture ratio and then cured for the needed duration. Following that, compressive strength can be determined by using the compressive test instrument. However, this strategy is time and money intensive, resulting in very low working efficiency. Unlike traditional experimental methods, certain empirical regression methods are offered to estimate concrete compressive strength using a given specified mixing ratio of different components in concrete. Unfortunately, the concrete mixture and compressive strength have a significant nonlinear relationship, making it challenging to obtain an adequate regression expression for this situation. The third technique to capture tangible behavior is through numerical simulation. The concrete matrix system presents a significant barrier in precisely predicting the compressive strength of concrete material^5–8^.

With the advancement of Artificial Intelligence (AI) in recent years, Machine Learning (ML) models have been widely used in various civil engineering industries, such as building materials, to assist with mix design and cement material performance optimization. ML is a subset of AI that can be used for a variety of tasks, including classification, regression, and clustering. ML models perform better in massive data analysis, where different ML models demonstrate their appropriateness for a wide range of data types because of their outstanding capacity to recognize complicated and unpredictable relationships between input and output dataset characteristics. Predicting concrete compressive strength is just one use of machine learning’s regression function. Compared to other traditional regression methods, ML algorithms learn directly from input data themselves and deliver extremely accurate results for the output data, demonstrating a clear advantage over the old regression methods^9–13^.

Several successful machine learning models, such as tree-based ensembles, support vector machines, artificial neural networks, multivariate adaptive regression splines, and others, can simulate a wide range of advanced technical properties of material composites. In large data sets for compressive strength prediction, a few popular ML models, such as Extreme Gradient Boosting (XGBoost), random forest, and hybrid ML models, are commonly used. ML models are widely used to forecast the mechanical properties of concrete. These strategies use a large amount of data to create a precise model. Their prediction accuracy is determined by the data sample utilized in experimental work during specimen casting or by a literature review. Researchers utilize these algorithms to predict the mechanical properties of concrete. Several studies have predicted the mechanical properties of numerous newly developed advanced concrete types, including self-healing concrete, recycled aggregate concrete, fiber-reinforced rubberized recycled aggregate concrete, high-strength concrete, and ultra-high strength concrete. Javed et al. forecast the compressive strength of sugarcane bagasse ash concrete using gene expression programming. The author used the experimental test to calibrate and validate the model^14–26^.

A major challenge in cement production is its significant contribution to greenhouse gas emissions. To support sustainability and circular economy principles, recent research has explored environmentally friendly alternatives for concrete production. Studies have highlighted the potential of incorporating oil palm by-products into sustainable lightweight structural concrete (SLSC), offering benefits in lifecycle performance, land-use efficiency, and cost-effectiveness. One study developed predictive models for the compressive strength of SLSC using advanced machine learning techniques, based on a dataset of 449 experimental samples. Among these, the MARS model demonstrated superior predictive accuracy. Key influencing factors were identified as gravel content, oil palm waste aggregate, and water-to-binder ratio. The research also included a Monte Carlo simulation and parametric analysis to validate and strengthen the reliability of the proposed models, showcasing the potential of ML-based approaches for strength prediction in sustainable concrete design^27^.

Advanced machine learning approaches are explored to improve the prediction of concrete compressive strength, a critical factor in structural design and durability assessment. One notable study introduced a hybrid ensemble model (HENSM) that combines multiple conventional machine learning techniques—ANN, linear and nonlinear MARS, GPR, and MPMR—using an additional ANN layer to enhance predictive accuracy. Based on a dataset of 1,030 entries from the UCI Machine Learning Repository, the study demonstrated that the ensemble model outperformed individual models in both training and testing phases. The results suggest that such hybrid methods not only improve prediction accuracy but also help mitigate overfitting, making them promising tools for sustainable concrete design and performance forecasting^28^.

Non-destructive testing (NDT) techniques, such as ultrasonic pulse velocity and the Schmidt rebound hammer test, are widely used due to their simplicity, speed, and efficiency. However, these methods often produce results with high variability and can significantly deviate from actual compressive strength values. To address this limitation, previous studies have explored the use of artificial neural networks (ANNs) to improve strength prediction accuracy. By training ANNs on experimental data from NDT methods, researchers have shown that these models can closely match true compressive strength values. One such study demonstrated the robustness and reliability of ANNs for this purpose, even providing model parameters for practical implementation, such as in spreadsheets for wider accessibility^29^.

A recent study investigated the effects of different sand gradings on the compressive strength of cement grout modified with a water-reducing polymer, following ASTM and BS standards. The research examined five sand types and polymer dosages up to 0.16% of cement weight, observing significant improvements in both fresh and hardened grout properties. The water-to-cement ratio was reduced by up to 54.1% without compromising workability. Notably, coarser sands yielded higher strength at lower w/c ratios, while finer sands performed better at higher w/c ratios. The polymer addition enhanced compressive and cylindrical strength by over 100% in some cases, due to gel formation that reduced voids and increased density. The British Standard-based mixes showed up to 71% greater compressive strength than those made to ASTM specifications, and finer sands consistently delivered superior flexural performance^30^.

To address the environmental burden of nonbiodegradable polymer waste, recent research has focused on producing sustainable bricks incorporating cement, fly ash, M sand, and recycled polypropylene fibers. This study employed advanced machine learning models—ANN, SVM, Random Forest, and AdaBoost—to predict the compressive strength of these eco-friendly bricks. By using SHAP (SHapley Additive exPlanations), the authors enhanced model transparency, identifying key input variables like age and fly ash as significant predictors. Among the models tested, ANN and Random Forest delivered the highest accuracy, with ANN achieving R² values above 0.99 and minimal RMSE. The work demonstrates both the effectiveness of machine learning in forecasting material properties and the value of explainable AI in sustainable construction^31^.

Kumar et al. focuses on predicting the compressive strength of ultrahigh performance concrete (UHPC) using advanced machine learning techniques to overcome the limitations of traditional statistical approaches in handling nonlinear relationships. A dataset containing 15 input variables related to mixture composition and aggregate characteristics was used to train several models, including the group method of data handling, recurrent neural networks, LSTM, and Bi-LSTM. Among these, the Bi-LSTM model achieved the best performance, with an R² of 0.9464 and RMSE of 0.0482 during testing. The findings highlight the model’s potential to optimize material selection, reduce experimental efforts, and lower development costs in UHPC mix design^32^.

Also, Satyanarayana et al. investigates the seismic performance of reinforced concrete T-beam bridges equipped with elastomeric bearings. The analysis incorporates region-specific ground motions to evaluate seismic vulnerability, with fragility curves used as the primary tool for quantifying failure probabilities across different loading intensities. To address uncertainties and improve prediction accuracy, the study applies artificial neural networks (ANN) and long short-term memory (LSTM) models, linking structural characteristics to fragility parameters. The findings support the development of precise fragility curves and enhanced risk assessments for bridge structures during seismic events^33^.

The research structure is organized as follows: Methodology that shows the study aim and the ML models used. Data that describes the data set used in the study. Statistical Analysis for the data. Machine Learning Models that describe a brief on each model used. Results and Discussion that include the analysis of results.

## Research significance

Conventional methods for estimating concrete compressive strength often rely on fixed equations or basic statistical tools. While useful in some cases, these approaches can fall short when dealing with the complex and nonlinear nature of real-world concrete mixes. They may not fully account for how multiple ingredients interact or how changes in proportions affect strength. This study aims to bridge that gap by using machine learning models that can better handle these complexities. By exploring and comparing several ML techniques, the research provides a more flexible and accurate alternative to traditional prediction methods.

By testing a variety of regression models—from simple linear ones to more advanced algorithms like XGBoost—this research highlights how ML can be a powerful tool in concrete design. The results not only show which models perform best but also support the broader use of data-driven techniques in civil engineering. These insights can help reduce testing costs, streamline the mix design process, and lead to more reliable concrete structures.

One key limitation in ML is sensitivity to data distribution as ML models often perform well within the range of training data but may struggle to extrapolate beyond it, especially if the dataset does not fully represent all practical mix design scenarios. Additionally, model generalizability can be limited if the training data is imbalanced or lacks diversity in material properties, environmental conditions, or curing practices. To mitigate this, we applied cross-validation and carefully analyzed feature distributions to ensure reasonable coverage. However, we recognize that the model’s performance may vary when applied to external datasets or real-world settings.

## Methodology

The basic dataset includes variables including cement content, silica fume, water, superplasticizer, sand, gravel, and curing age. The goal variable is compressive strength, which is measured in megapascals (MPa). The dataset was preprocessed to remove missing values, standardize the features, and separate the training and testing sets.

Various regression models were used, including Linear Regression, Ridge, Lasso, Decision Trees, Random Forest, SVR, XGBoost, and KNeighbors Regressor. To optimize the models, hyperparameters were tuned, and measures such as Mean Absolute Error (MAE), Mean Squared Error (MSE), and R-squared were used for evaluation.

### Data

The dataset we used comprises 167 concrete mix records, each with multiple input features including age, water content, cement, aggregates, and admixtures, see supplementary file. We acknowledge the importance of ensuring the dataset captures a representative range of parameter values for robust model generalization. To assess coverage, we compared the input value distributions with ranges reported in relevant literature. These references detail experimentally validated ranges for common concrete constituents and mix designs. We found that our dataset spans a comparable, and in several cases broader, range. Each observation consists of the following features:

- Cement (kg/m^2^): 400–600.
- Silica Fume (kg/m^2^): 0–100.
- Water (kg/m^2^) varies between 108 and 180.
- Superplasticizer (kg/m^2^) ranges from 0 to 20.
- Sand (kg/m^2^) ranges from 400 to 800.
- Gravel (kg/m^2^): 900–1300.
- Age (days): The curing time of the concrete sample, which ranges between 3 and 56 days.
- Compressive Strength (MPa): The strength of the concrete sample, measured in MPa. Before training the machine learning models, the data needed to be adjusted to a consistent scale so that all features could be fairly considered during the learning process. Since the dataset included parameters with different measurement units and value ranges, normalizing the data helped prevent any one feature from overpowering the others. To do this, the values in each feature were scaled between 0 and 1 by dividing each value by the maximum. Once normalized, the data records were shuffled to remove any order-related bias and then split into two sets: 70% of the data was used to train the models, while the remaining 30% was set aside to test how well the models could predict new, unseen results. This approach helped ensure that the models were both accurate and reliable.

### Statistical analysis

**Fig. 1:**
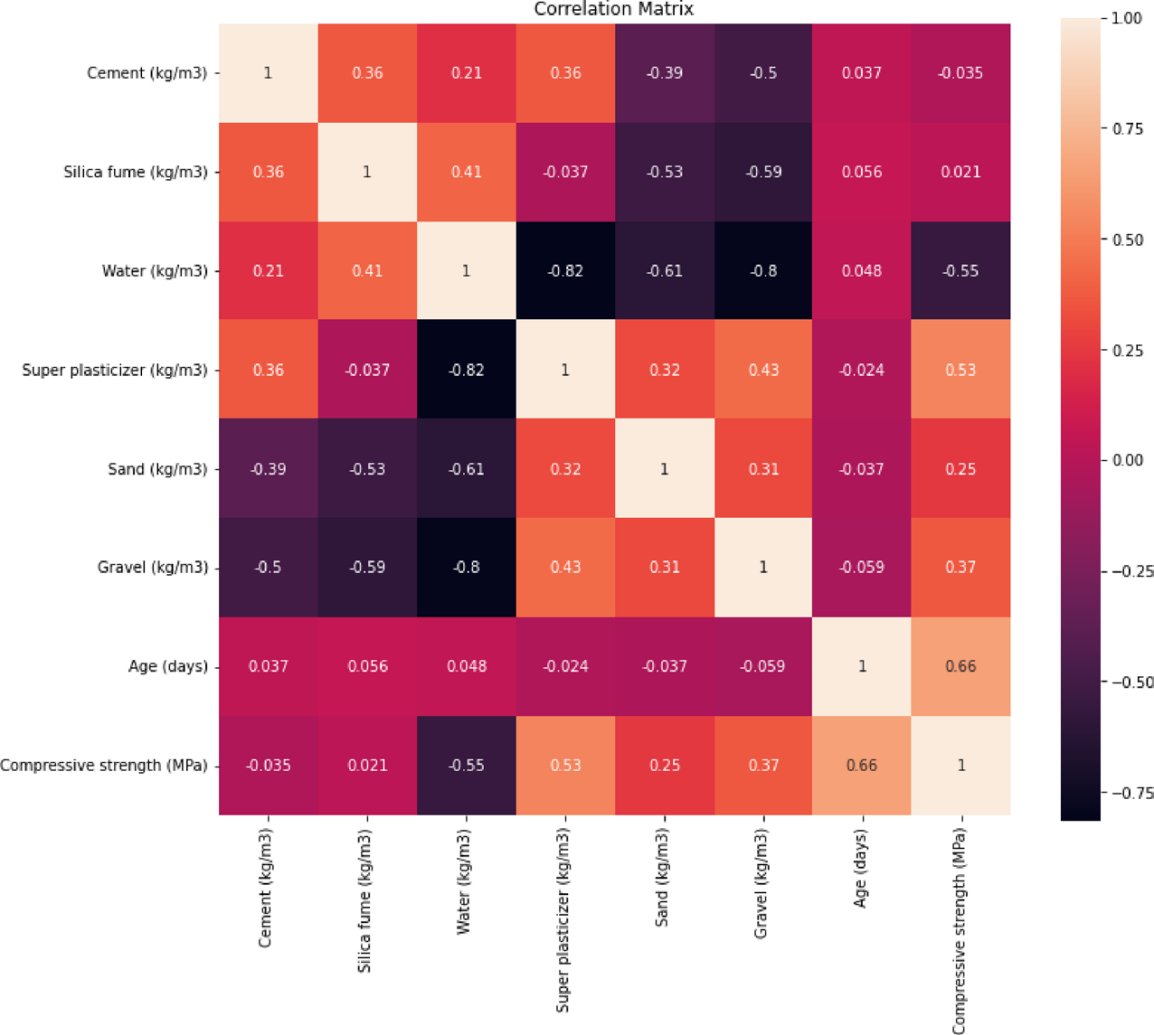
Correlation matrix for the data inputs.

The initial statistical analysis of the dataset revealed variable connections between the input features and the compressive strength of concrete. The correlation matrix demonstrated that water and gravel have a negative association with compressive strength, whereas superplasticizer had a positive correlation as shown in Fig. 1. The water-to-cement ratio plays a critical role in compressive strength, as higher water content decreases the strength. Superplasticizers help increase the strength by reducing the amount of water needed in the mix. The results also show that the age of the concrete is a major factor, with older samples showing higher compressive strength.

From the statistical summary that can be shown in Table 1:

- Mean Compressive Strength: 59.93 MPa.
- Cement: The cement content showed a weak correlation of − 0.035 with compressive strength.
- Water: Strong negative correlation of − 0.55 with compressive strength.
- Superplasticizer: Positive correlation of 0.53 with compressive strength.

**Table 1:** Statistical analysis of the data.

	Cement (kg/m3)	Silica fume (kg/m3)	Water (kg/m3)	Super plasticizer (kg/m3)	Sand (kg/m3)	Gravel (kg/m3)	Age (days)	Compressive strength (MPa)
Mean	460.396	82.27723	154.2871	11.42822	599.851	1126.366	22.683	59.93069
Std	40.81472	29.51927	39.00778	5.065858	62.3928	97.2116	20.9164	17.39764
MIn	400	0	108	0	443	961	3	28
Max	600	120	243	18	709	1329	56	91.2

**Fig. 2:**
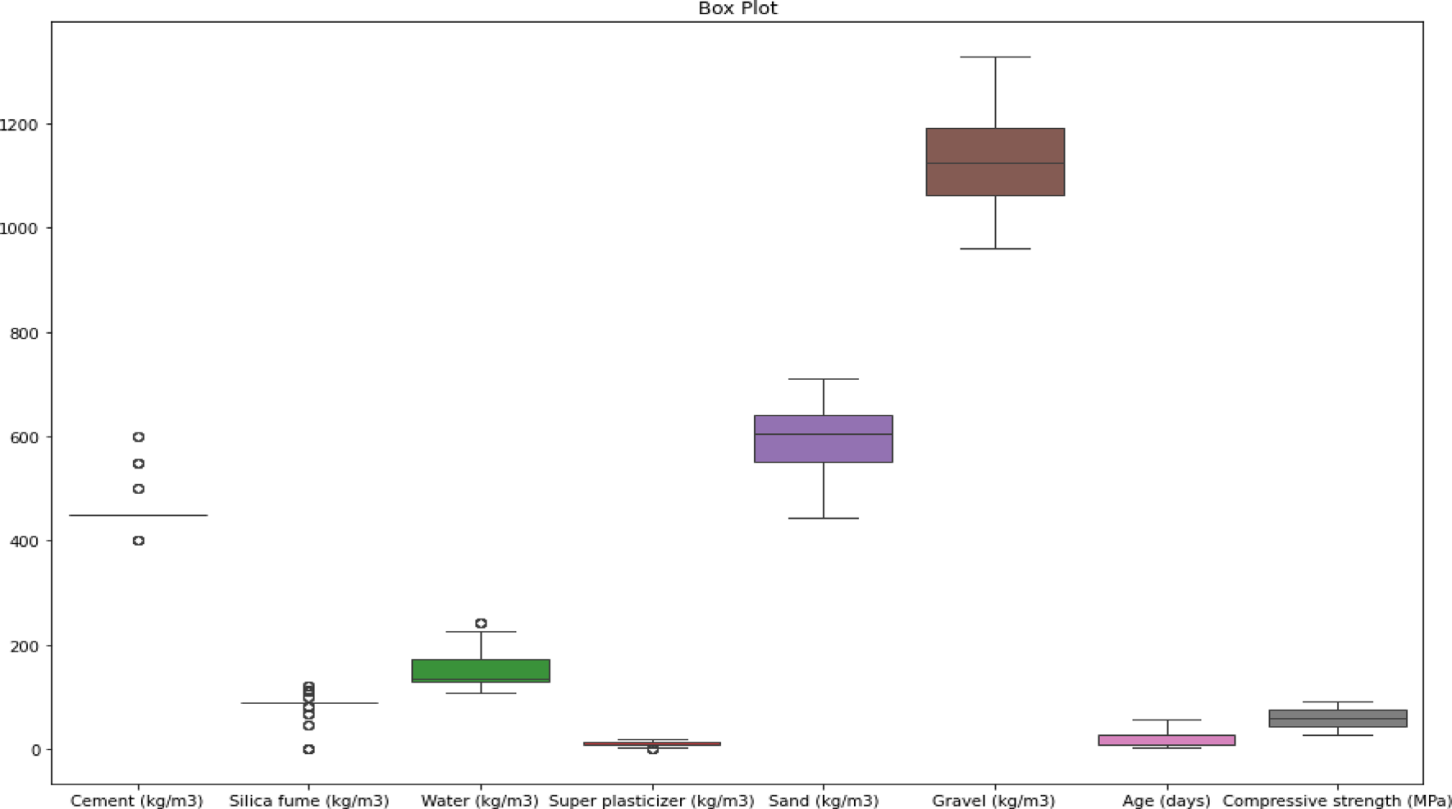
The Box plot for the data used.

Figure 2 illustrates that variables like cement, sand, and water have moderate variability, whereas gravel has the broadest spread. Age and Superplasticizer have the least variation. Compressive strength has a tight distribution, showing that most blends attained comparable strength, albeit a few samples performed poorly. These visualizations can be used to better comprehend the range and consistency of each material’s application, as well as its impact on compressive strength results.

### Machine learning models summarization

Multiple ML models were applied, including:

####  Linear regression

Linear Regression is a basic ML approach that predicts a continuous target variable by creating a linear relationship between input data and output. To fit a line to the data, it minimizes the sum of squared residuals (the difference between the observed and predicted values). However, because it presupposes a linear relationship between variables, it may be ineffective when dealing with non-linear data.

####  Ridge regression

Ridge Regression is a regularized variant of Linear Regression that includes a penalty term proportional to the square of the magnitude of the coefficients in the loss function. This strategy helps to reduce overfitting, particularly when dealing with multicollinearity or a high number of predictors. The regularization term reduces the model coefficients, resulting in more generalizable models.

#### Lasso regression

Lasso (Least Absolute Shrinkage and Selection Operator) Regression penalizes the sum of absolute coefficient values, resulting in sparse solutions with some feature coefficients shrunk to zero. This property makes Lasso useful for feature selection because it automatically removes unnecessary variables, enhancing model simplicity and understanding while simultaneously addressing overfitting.

#### Decision tree

Decision Tree is non-parametric models that divide a dataset into subsets based on input features in order to predict a target variable. They are interpretable and can handle categorical and numerical data. However, decision trees are prone to overfitting, particularly when grown to full depth, and their forecasts might vary greatly.

#### Random forest

Random Forest is an ensemble method for creating numerous decision trees from bootstrap samples and random feature selection. The final forecast is obtained by averaging the outputs of the individual trees (for regression). This technique avoids overfitting and improves robustness when compared to single decision tree, making Random Forest a dependable model for a variety of regression situations.

#### SVR

SVR is a strong regression technique based on Support Vector Machines that predicts continuous outcomes by identifying the function that best fits the data within a given margin of tolerance (epsilon). It handles non-linear interactions using kernel functions such as linear or radial basis functions (RBF), and its margin-based approach makes it resistant to outliers. SVR is commonly employed in applications that need high prediction accuracy, such as financial forecasting and time series analysis.

#### XGBoost (extreme gradient boosting)

XGBoost is an efficient and scalable gradient boosting method that trains several weak learners (usually decision trees) sequentially to rectify earlier model mistakes. It employs advanced techniques including regularization, parallel processing, and early pausing to improve model performance and reduce overfitting. XGBoost is well known for its excellent predicted accuracy and is widely employed in both competitions and real-world applications.

####  KNeighbors regressor

The KNeighbors Regressor is a straightforward, instance-based method that predicts based on the average of the target values of the k-nearest neighbors in the training data. It assumes that similar situations result in similar outcomes. The model’s performance is largely dependent on the value of k and the distance metric, and it may struggle with high-dimensional data or excessive noise in the features.

## Results and discussion

**Table 2:** Results of ML models.

Model name	Mean *R*-squared (R^2^)	Mean squared error (MSE, MPa)	Mean absolute error (MAE, MPa)
Linear regression	0.688103	96.3833	7.629474
Decision tree	0.881777	36.53361	3.880556
Random forest	0.920541	24.55467	3.735522
Ridge	0.70731	90.4479	7.437402
Lasso	0.622144	116.7662	9.321875
SVR	0.846786	47.34675	5.004105
KNeighbors regressor	0.723216	85.53269	7.272222
XGB regressor	0.940764	18.30525	2.605828

**Fig. 3:**
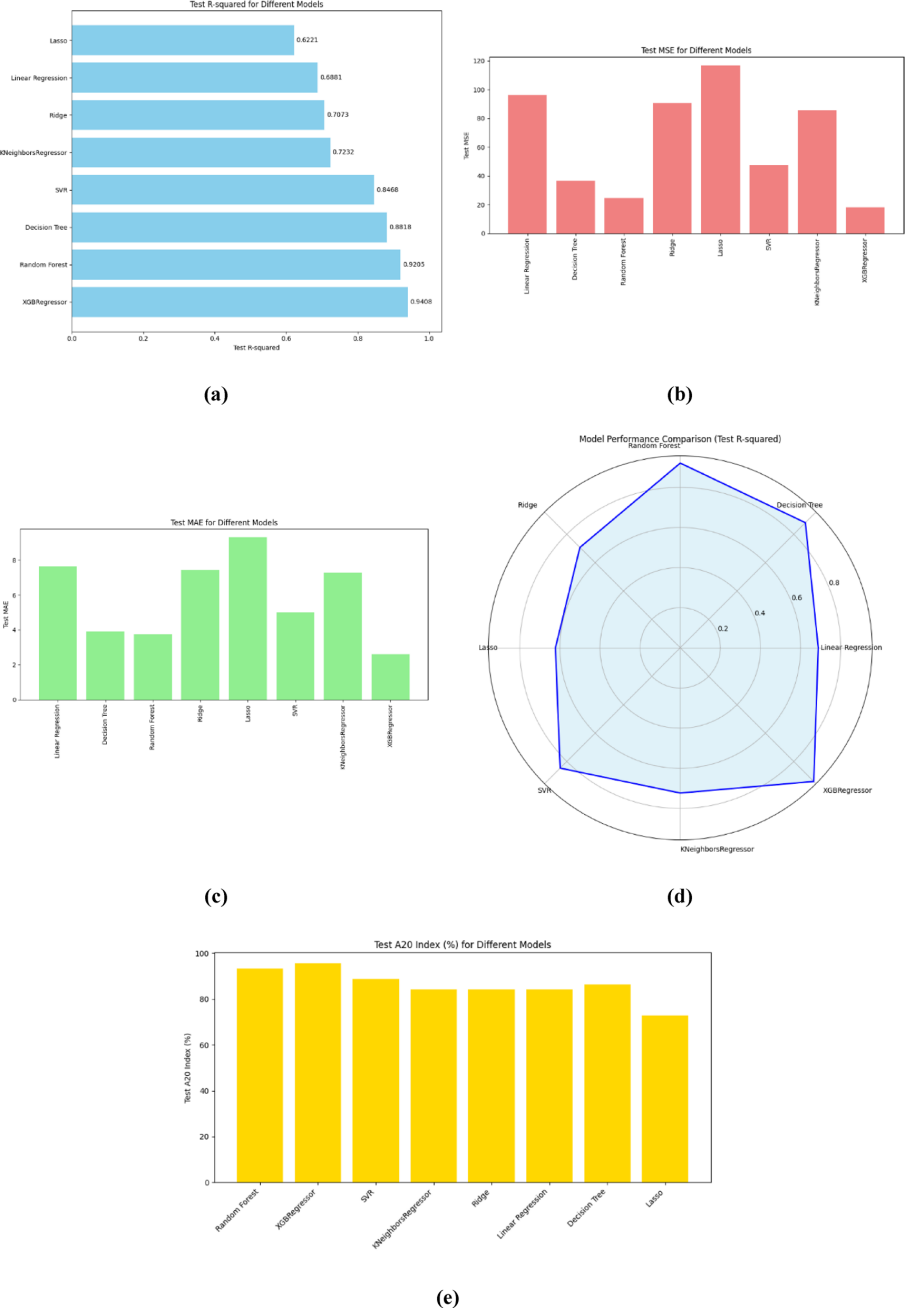
ML models evaluation metrics.

To mitigate overfitting in our proposed model, we employed several best practices. The dataset was split into training and test sets using stratified sampling where applicable, and model performance was evaluated using cross-validation to ensure generalizability across different data subsets. Hyperparameter tuning was performed using grid/random search with cross-validation to avoid overly complex model configurations. Additionally, we monitored both training and validation errors to ensure alignment and prevent memorization of the training data^34^.

Table 2; Fig. 3 show values and charts that compare the effectiveness of the used ML models on predicting concrete strength using three metrics: Mean Absolute Error (MAE), Mean Squared Error (MSE), and R-squared (R²). A radar plot is also included to demonstrate model comparisons.

Figure 3a shows the Mean R-squared that calculates the proportion of variation in the dependent variable that is predictable from the independent variables. Higher R² suggests improved performance. XGBoost has the greatest R-squared value (~ 0.94), indicating that they account for the largest variance in compressive strength predictions. Linear Regression and Lasso have lower R-squared values, indicating that they explain less of the variability in the target variable. Decision Trees perform reasonably well; however, they lag somewhat below ensemble approaches.

Figure 3b shows the MSE (mean squared error). XGBoost once again outperforms with the lowest MSE, which means its squared prediction errors are minimized. Lasso has the greatest MSE, indicating that it performs the poorest according to this metric, while Random Forest and KNeighbors Regressor both perform better. A lower MSE indicates more accurate models, as larger errors are penalized more severely.

Figure 3c shows the Mean Absolute Error (MAE), and from this figure it is observed that XGBoost has the lowest MAE, which means it makes the fewest errors on average when predicting compressive strength. Random Forest also performs well, but Lasso has the greatest MAE, indicating that it makes the most prediction errors. Lower MAE values are desired because they indicate more accurate predictions, making XGBoost and Random Forest the better options based on this statistic.

Figure 3d shows the radar chart that shows a visual comparison of models based on numerous performance indicators. XGBoost, Random Forest, and Decision Tree have the largest regions in the plot, indicating greater performance in terms of prediction accuracy and variance explained (R-squared). Linear regression and Lasso cover smaller areas, implying that they are less effective at predicting concrete compressive strength. KNeighbors Regressor performs moderately across measures, but lags ensemble approaches like XGBoost and Random Forest.

Figure 3e shows the A20 index chart that illustrates the percentage of predictions within ± 20% of the true values for various regression models. Among all models, XGBoost achieved the highest A20 accuracy, indicating a strong alignment between its predictions and actual outcomes. Random Forest and SVR also performed robustly, closely trailing XGBoost. In contrast, Lasso Regression exhibited the lowest A20 score, suggesting that a greater proportion of its predictions deviated significantly from true values. Overall, ensemble and kernel-based methods demonstrated superior reliability in maintaining predictive accuracy within acceptable error margins.

Based on the evaluation metrics, XGBoost is the best-performing model across all criteria, followed closely by Random Forest, both of which excel at reducing mistakes and explaining variance in compressive strength predictions. Lasso and Linear Regression perform poorly when compared, particularly in terms of MAE and MSE. The radar graphic clearly shows that ensemble models routinely outperform simpler methods such as Linear Regression and KNeighbors Regressor.

**Fig. 4:**
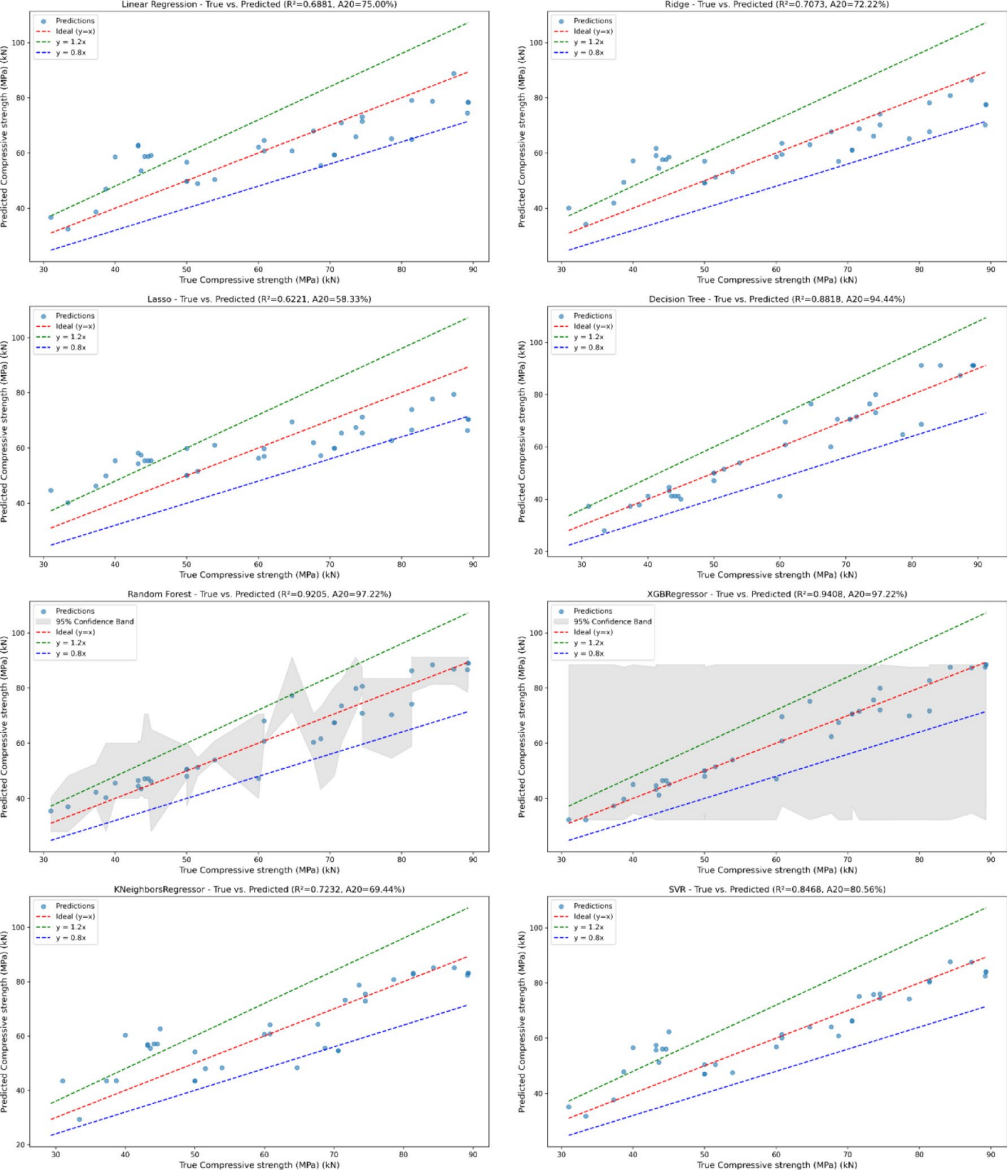
Ture vs. predicted for all used ML models.

The scatter plots shown in Fig. 4 represents the link between true and predicted compressive strength values for different ML models. The red dashed line shows the ideal relationship, in which projected values exactly match true values (a 1:1 correlation). The plots show that the Decision Tree and SVR model with R-squared = 0.8818 and 0.8468, respectively predicts well, with most points falling near the line of perfect prediction. However, certain points deviate, particularly at higher real response values, indicating that the Decision Tree has difficulty with larger compressive strengths.

The K-Neighbors Regressor with R-squared = 0.7232 exhibits greater divergence from the ideal line, particularly for lower and higher genuine response values. This shows that the KNeighbors Regressor has trouble accurately forecasting compressive strength across a wide range of values.

Lasso Regression model with R-squared = 0.6221 displays a moderate spread around the optimal line. Many predictions fail, especially at greater compressive strengths, showing that the model is not successfully capturing all data correlations.

Linear Regression model with R-squared = 0.6881 outperforms Lasso but still deviate significantly from the perfect prediction line. The model underpredicts or overpredicts various variables, particularly at the extremes of the range.

Random Forest model with R-squared = 0.9205 works admirably, with most projected values closely matching the true values. This ensemble model efficiently represents the data’s non-linear interactions with little variation from the ideal line.

Ridge Regression model with R-squared = 0.7073 performs reasonably well but has a moderate spread around the optimum forecast line. Higher compressive strengths cause underestimation, but it outperforms Lasso and Linear Regression.

XGBoost model with R-squared = 0.9408 works admirably, with projected values firmly grouped around the ideal line. Its ensemble approach enables it to detect complicated patterns in data, resulting in extremely accurate predictions.

### Effect of SHAP value on model output for concrete composition

**Fig. 5:**
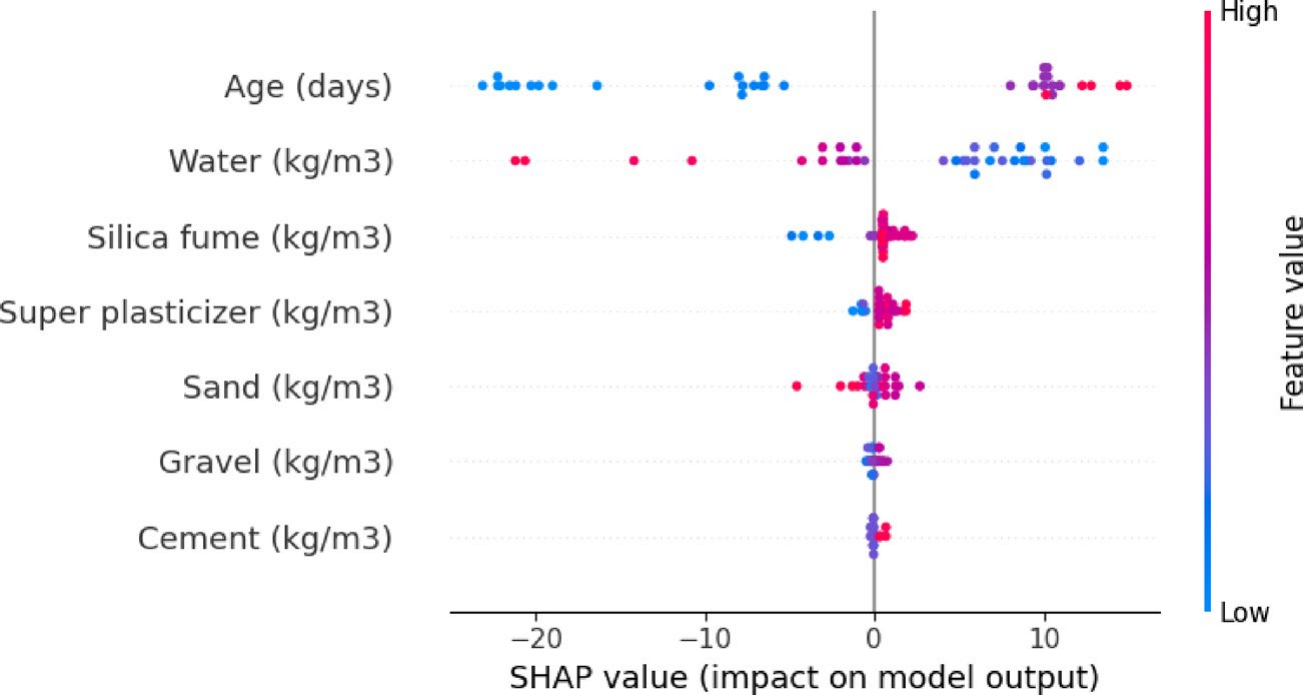
SHAP value on model output.

Figure 5 The SHAP analysis highlights Age (days) as the most influential feature positively affecting the model output, which likely corresponds to concrete compressive strength. This aligns with the fundamental chemistry of cement hydration: as curing progresses over time, more calcium silicate hydrate (C-S-H) gel forms, densifying the matrix and significantly enhancing strength. Particularly in the early stages (e.g., 1–28 days), strength gain is rapid, and the continued hydration process extends benefits over longer periods, explaining Age’s dominant SHAP impact. Gravel (kg/m³), on the other hand, exhibits a relatively minor influence. This is expected, as gravel primarily serves as a coarse aggregate providing volume stability rather than directly participating in chemical reactions. Its contribution to strength is more structural and indirect, mainly influencing workability and the aggregate-paste interfacial transition zone (ITZ), which typically has less variation in a well-designed mix. Hence, its role is less dynamic compared to reactive components like cement or admixtures, which directly modify the microstructure.

### GUI tool

**Fig. 6:**
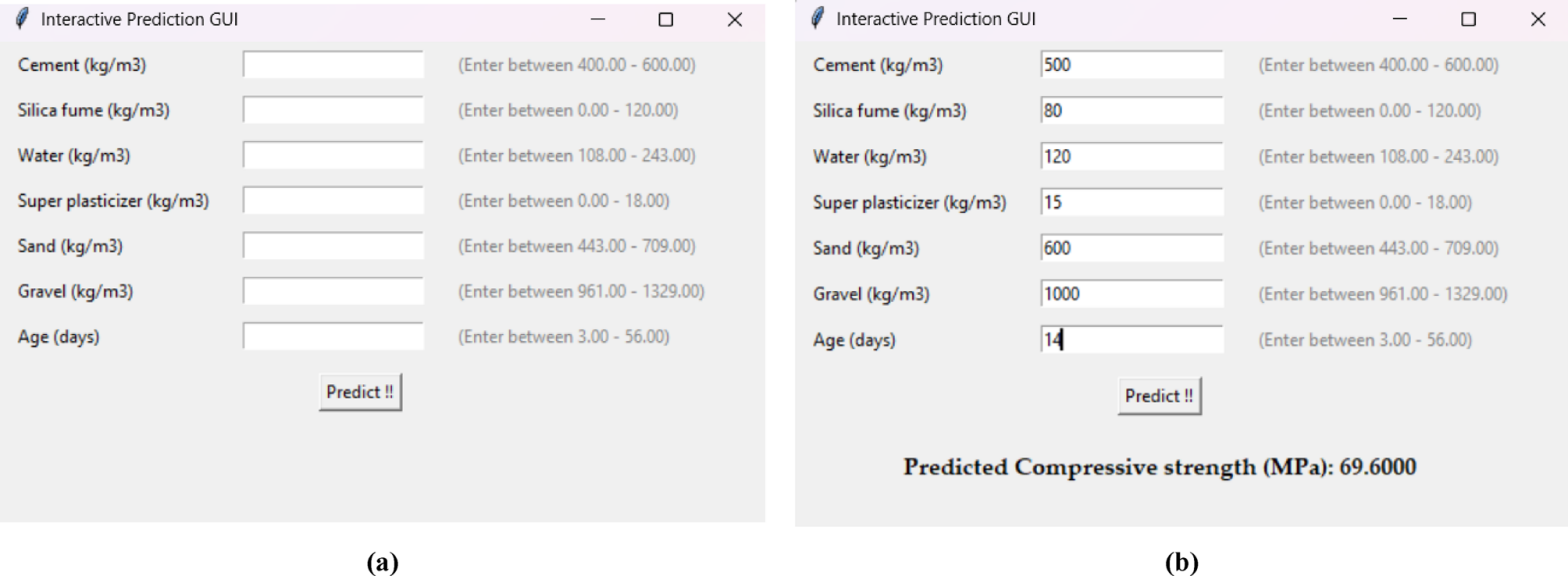
GUI tool. (**a**) The input interface. (**b**) The result.

To enhance the usability and practical application of the developed machine learning model, the authors created an interactive graphical user interface (GUI) tool^35^, as shown in Fig. 6. This user-friendly interface allows engineers and practitioners to input key concrete mix parameters—such as cement, silica fume, water, superplasticizer, sand, gravel, and age—within specified valid ranges. Once the inputs are provided, the tool instantly predicts the expected compressive strength using the trained model. This GUI eliminates the need for coding expertise, making advanced ML predictions accessible to a broader audience in the construction and materials engineering field. Such tools bridge the gap between model development and real-world implementation, supporting data-driven decision-making in concrete mix design.

## Conclusion

This study evaluated the performance of several machine learning algorithms in predicting the compressive strength of concrete based on its mix constituents. Among the models tested, XGBoost and Random Forest achieved the highest prediction accuracy (R² ≈ 0.94) and the lowest error metrics (MAE and MSE), demonstrating superior ability to model complex, non-linear relationships. These ensemble-based methods consistently outperformed simpler linear models such as Linear Regression, Lasso, and Ridge Regression, which struggled to capture the intricate interactions among variables. The results highlight the value of incorporating advanced ML techniques in structural engineering, particularly for applications where traditional models fall short. Ensemble models offer robust and reliable predictions, making them practical tools for aiding material design and quality control decisions. Lastly, integrating these predictive models into user-friendly graphical user interface (GUI) tools could greatly enhance accessibility for practitioners. Such tools would allow engineers to input mix design parameters and quickly obtain strength predictions, facilitating informed decision-making without requiring deep ML expertise.

For future work, applying feature engineering strategies—such as polynomial expansions, interaction terms, or dimensionality reduction—could further enhance model performance. Additionally, expanding the dataset to include environmental parameters like curing temperature and humidity could improve generalization and prediction accuracy. Testing the trained models on external datasets is also recommended to assess their transferability.

## Electronic supplementary material

Below is the link to the electronic supplementary material.

Supplementary Material 1.

## Data Availability

All data generated or analyzed during this study are included in this published article as supplementary information files. In addition, any additional required data is available by the corresponding author.

## Abbreviations

HSC: High strength concrete
AI: Artificial intelligence
ML: Machine learning
MAE: Mean absolute error
MSE: Mean squared error
XGBoost: Extreme gradient boosting
SLSC: Sustainable lightweight structural concrete
ANN: Artificial neural network
NDT: Non-destructive testing
UHPC: Ultra-high-performance concrete
SVR: Support vector machine
ITZ: Interfacial transition zone
